# Supplementary material for: Comparison of Feeding Efficiency and Hospital Mortality between Small Bowel and Nasogastric Tube Feeding in Critically Ill Patients at High Nutritional Risk
Source: Nutrients. 2020 Jul 6;12(7):2009. doi: 10.3390/nu12072009 (PMC7400848; doi:10.3390/nu12072009)
Supplement: Supplementary file 1 [file nutrients-12-02009-s001.pdf]

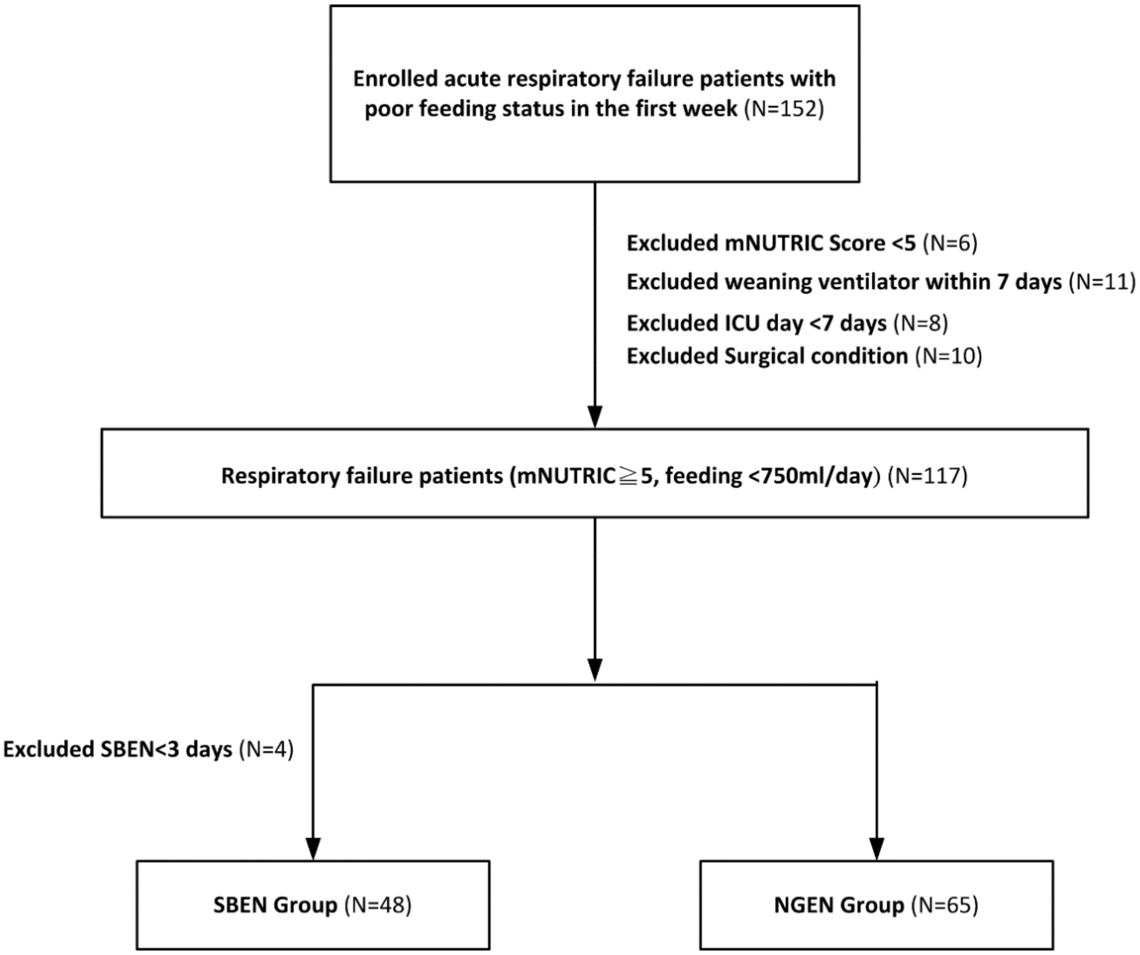

Figure 1. Study flow chart. "SBEN group" was defined as "Feeding by small bowel enteral nutrition at the 7th ICU day". "NGEN" was defined as "Keep feeding by nasogastric tube enteral nutrition at the 7th ICU day".
